# Opening the black box: explainable deep-learning classification of wood microscopic image of endangered tree species

**DOI:** 10.1186/s13007-024-01191-6

**Published:** 2024-04-24

**Authors:** Chang Zheng, Shoujia Liu, Jiajun Wang, Yang Lu, Lingyu Ma, Lichao Jiao, Juan Guo, Yafang Yin, Tuo He

**Affiliations:** 1 Department of Wood Anatomy and Utilization, Research Institute of Wood Industry, Chinese Academy of Forestry, Beijing, 100091 China; 2 Wood Collections, Chinese Academy of Forestry, Beijing, 100091 China; 3 Wildlife Conservation Monitoring Center, National Forestry and Grassland Administration, Beijing, 100714 China; 4 National Centre for Archaeology, Beijing, 100013 China

**Keywords:** Computer vision, Deep learning, Feature visualization, Image classification, Wood identification

## Abstract

**Background:**

Traditional method of wood species identification involves the use of hand lens by wood anatomists, which is a time-consuming method that usually identifies only at the genetic level. Computer vision method can achieve "species" level identification but cannot provide an explanation on what features are used for the identification. Thus, in this study, we used computer vision methods coupled with deep learning to reveal interspecific differences between closely related tree species.

**Result:**

A total of 850 images were collected from the cross and tangential sections of 15 wood species. These images were used to construct a deep-learning model to discriminate wood species, and a classification accuracy of 99.3% was obtained. The key features between species in machine identification were targeted by feature visualization methods, mainly the axial parenchyma arrangements and vessel in cross section and the wood ray in tangential section. Moreover, the degree of importance of the vessels of different tree species in the cross-section images was determined by the manual feature labeling method. The results showed that vessels play an important role in the identification of *Dalbergia*, *Pterocarpus*, *Swartzia*, *Carapa*, and *Cedrela*, but exhibited limited resolutions on discriminating *Swietenia* species.

**Conclusion:**

The research results provide a computer-assisted tool for identifying endangered tree species in laboratory scenarios, which can be used to combat illegal logging and related trade and contribute to the implementation of CITES convention and the conservation of global biodiversity.

**Supplementary Information:**

The online version contains supplementary material available at 10.1186/s13007-024-01191-6.

## Background

Illegal logging is the most profitable natural resource crime over the world. The financial value of illegal logging and related trade is approximately $52 to $157 billion per year [1]. Therefore, the international community has emphasized the Convention on International Trade in Endangered Species of Wild Fauna and Flora (CITES) to ban or restrict trade in endangered tree species to combat illegal logging and related trade [2–6]. As of 2023, approximately 670 tree species have been listed in CITES Appendices because of their overexploitation. The main barrier to the implementation of CITES is the definitive identification of traded timber and wood products, where forensic tools are urgently required [3, 7].

In the Neotropics (South and Central America and tropical Mexico), three commercially important species of *Swietenia* (Meliaceae) are listed in CITES Appendix II [8]. The wood of these three species are widely considered indistinguishable by wood anatomists [9, 10]. *Dalbergia* and *Pterocarpus* are two other important genera of Leguminosae, often referred to as rosewood tree species [11], and most species from these genera are threatened by illegal logging activities.

All *Dalbergia* species (except *D. nigra* which is listed in Appendix I) are listed in Appendix II, and for *Pterocarpus* spp., *P. santalinus*, *P. erinaceus*, *P. tinctorius* as well as *Pterocarpus* species that are from an African population are also listed in CITES Appendix II. These woods are used for furniture, musical instruments, and handcrafts because of their beauty, workability, and moderate resistance to corrosion, and are highly sought after by consumers worldwide [12, 13]. In the international trade of CITES-listed tree species, documents with fake names of similar species are often submitted to customs officials to avoid inspection. Consequently, the discrimination of CITES-listed tree species from their look-alikes is a key step in combating the illegal timber trade.

Wood anatomy is one of the most important methods for field wood identification [10, 14, 15], and is performed by observing various anatomical features using a hand lens in three orthogonal directions, i.e., cross, radial, and tangential [16]. However, wood identification is a difficult task that requires specialized anatomical knowledge and a wide range of interspecies and intraspecies similarities [17]. This results in professional wood anatomists often requiring decades of specialized training to achieve genus-level identification. In contrast, computer vision can provide an economical alternative to human-based biological domain support for in situ screening of wood in trade, which is faster, does not require individual skill training and can yield species-level identification if sufficient images covering intraspecific variation are available for model training.

Currently, computer vision is developing rapidly, and there has been a lot of work done in wood macro image classification [18–20]. In forensic wood identification, it is often necessary to provide identification keys, namely the features on which experts base their judgments. However, the deep-learning model is like a black box, which cannot provide the basis of judgment in classification as a wood anatomist can. Although many studies have demonstrated that wood anatomy images coupled with deep learning can discriminate between wood species and their look-alikes at the species level, the diagnostic features extracted by this model remain invisible. In the context of wood identification, wood anatomists are not only interested in what the species is, but also want to know what anatomical features can accurately and efficiently discriminate this species from their look-alikes.

Existing research has shown that vessel is the key feature for wood identification [19]. With the help of feature visualization, it can explore whether the key features recognized by intelligence methods are consistent with wood anatomists. Meanwhile, it is possible to explore the inherent features in wood species to determine the differences in wood anatomical features between similar species [21, 22]. Feature visualization will help wood anatomists to be more effective when conducting wood identification tasks in the field [19].

As two commonly used methods in computer vision, machine learning and deep learning have different workflows. Machine learning-based wood identification is an information-driven research field in which many researchers understand wood identification from a new perspective based on the knowledge of wood science. Machine learning methods require researchers to assemble a dataset by gathering wood anatomical, chemical, or genetic information and then analyze it using unsupervised or supervised models [23, 24]. This information can be collected in the form of images, videos, text, and measurements. Deep learning models can make use of image data to a greater extent over other types of data. Although deep-learning models can accurately identify wood species, interspecific differences in morphological features still need to be determined.

The purpose of this study was to reveal interspecific differences between similar tree species using computer vision methods. First, a dataset of slide images in the cross and tangential sections of 15 similar tree species was created and a deep-learning model was established. Feature visualization was then conducted to target the key differences between species in the image classification. Then, the degree of importance of the vessels in the cross sections of different wood species was determined using the manual feature labeling method. The model developed in this study provides a tool that can identify wood species quickly and visualize important features that can help anatomists complete identification work more accurately and support effective CITES implementation.

## Materials and methods

### Data preparation

Fifteen species from *Carapa*, *Cedrela*, *Dalbergia*, *Swartzia*, *Pterocarpus,* and *Swietenia* were selected for this study and divided into four groups based on their anatomical similarity (Table 1). Multiple wood specimens of the selected species were collected for sectioning [25] and 2–3 images of the cross-or tangential sections of the heartwood were collected for each specimen. Images of 4096 × 2160 pixels and 8-bit RGB in PNG format, representing 2.23 × 0.78 mm of tissue, were captured using a microscope at 5 × magnifications.

**Table 1:** Protection level, number of wood specimens, and collected images for selected species

Class label	Group	Species	Protection level	Number of Specimens	Image quantity		
					Cross section	Tangential section	Total
**1**	Group1	*Carapa guianensis*	–	7	30	30	60
**2**		*Cedrela fissilis*	CITES II	6	30	30	60
**3**		*Cedrela odorata*	CITES II	10	30	30	60
**4**	Group2	*Dalbergia latifolia*	CITES II	6	30	30	60
**5**		*Dalbergia nigra*	CITES I	6	30	30	60
**6**		*Dalbergia stevensonii*	CITES II	7	30	30	60
**7**		*Dalbergia tucurensis*	CITES II	6	30	30	60
**8**		*Swartzia madagascar*	–	5	25	25	50
**9**	Group3	*Pterocarpus indicus*	–	6	30	30	60
**10**		*Pterocarpus macrocarpus*	–	6	30	30	60
**11**		*Pterocarpus soyauxii*	CITES II	5	25	25	50
**12**		*Pterocarpus tinctorius*	CITES II	5	25	25	50
**13**	Group4	*Swietenia humilis*	CITES II	9	25	25	50
**14**		*Swietenia macrophylla*	CITES II	15	30	30	60
**15**		*Swietenia mahagoni*	CITES II	12	25	25	50

A total of 50–60 images per species were captured to cover the variability of the tree species as much as possible and ensure data balance for each species. Details of data collection for the selected tree species are listed in Table 1 and their anatomical features of each group are presented in Additional file 1: Table S1. The images of *Carapa guianensis*, *D. latifolia,* and *P. indicus* were captured at both 2.5 × and 5 × magnifications to verify whether the visualization results were altered with image magnifications.

#### Image dataset construction and processing

Putting the original image of 4096 × 2160 pixels directly into the CNN model increases the burden of model training; thus, the patch sizes of 600 × 600, 800 × 800, 1000 × 1000, 1200 × 1200, 1400 × 1400, 1600 × 1600, 1800 × 1800, and 2000 × 2000 pixels were extracted from the original image to increase the size of the dataset [19], and a 20% repetition rate was left when the patches are extracted to ensure feature integrity. The extracted patches were divided into training and testing sets in a ratio of 8:2 and then fed into the deep-learning model ResNet152 for training, and the optimal test results were obtained after tuning the parameters. In terms of parameter tuning, the parameters such as learning rate, learning rate update strategy, image enhancement method, and maximum number of iterations were modified.

#### Vessel dataset introduction and processing

**Fig. 1:**
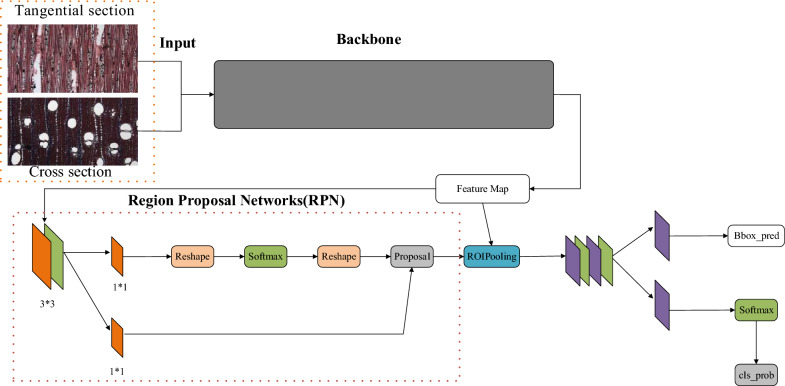
Faster R-CNN network model diagram, consisting of backbone and RPN network

The classification of individual features of wood microscopic images by manual annotation is typically performed using an object-detection model that contains backbone networks, neck networks, detection heads or other components, such as YOLO (You Only Look Once) [26], SSD (Single Shot MultiBox Detector) [27] and Faster R-CNN (Region Convolution Neural Network) [28]. The network structure of Faster R-CNN is shown in Fig. 1.

To test the effect of the vessel on the accuracy of the trained model, it is necessary to ensure that the original variables are fixed, and only the vessel features of wood are input. Object detection was performed mainly by building a ResNet152 (consistent with the wood classification model), with the addition of a neck network and the inclusion of a detection head (e.g., YOLO Head) used to regress the bounding box. In the regression of the bounding box, although the same backbone network (ResNet152) was used for object detection, a neck network such as the detection head (or RPN) would affect the detection results, which could not result in the quantitative analysis.

The labeling tool LabelImg [29] was used to label vessels in the images, to eliminate the influence of, for example, the neck network and the detection head. In the vessel dataset, the labeled vessels were directly cropped by means of image cropping to create a new image, which eventually composed a dataset of vessels of different tree species, and then, the same neural network model was used to train and test the vessel dataset to obtain the final classification results. Thus, the process was simpler, and no remodeling was required. The classification was more accurate and the influence of other network structures on the result eliminated, which fundamentally solved the problem of incorrect detection.

The vessel dataset contained vessels from cross-sectional images of the 15 tree species, and the details of the vessel dataset are shown in Additional file 2: Table S2. To balance the data, the dataset was processed before training, and for tree species with more vessel features, such as *Carapa guianensis* (455 vessel features) and *Swartzia madagascar* (798 vessel features), excessively duplicated vessels were removed. In addition, incomplete feature shots were inevitably present in the original images. Therefore, vessels with fewer than 50% missing areas were selected for retention.

### ResNet152 model architecture

**Fig. 2:**
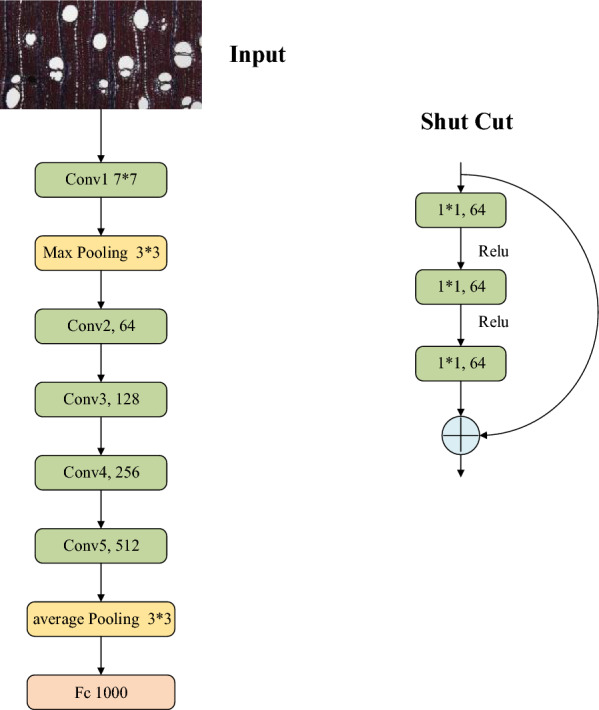
ResNet Network Model Diagram

Research has shown that in deep-learning models, the deeper the network layer, the higher the accuracy of the model. ResNet [30] is currently one of the best-performing neural networks for image classification tasks. The ResNet network structure mainly refers to VGG19 and adds residual units on top, which solves the degradation problem that occurs with the deepening of the network models. ResNet152 was used for training and testing, thereby laying the foundation for feature visualization. The network structure of ResNet152 is shown in Fig. 2.

The residual units consist of three convolutional kernels (1 × 1, 3 × 3, and 1 × 1) and a jump connection, which can be expressed as Formula 1:

1 $$y_l = h(x_l ) + F(x_l ,w_l )$$

2 $$x_{l + 1} = f(y_l )$$

where $x_l$ and $x_{l + 1}$ are the input and output of the lth residual unit, respectively, and each residual unit contains a multilayer structure, as shown in Fig. 2. $F$ represents the residual network, $h(x_l ) = x_l$ is the identity mapping, and $f$ is the ReLU (Rectified Linear Unit) activation function, which can be represented by Formula 3, where $x$ represents the input data.

3 $$f(x) = \left\{ {\begin{array}{*{20}c} {x,} \\ {a(e^x - 1),} \\ \end{array} } \right.\begin{array}{*{20}c} {x > 0} \\ {x \le 0} \\ \end{array}$$

### Training and prediction

The deep-learning model was trained using the training set and tested at each epoch using the test set. All patches were adjusted to 224 × 224 pixels for a total of 3 channels, and the average value of each channel was calculated from the entire image in the two datasets. A total of 400 patches were input into the model for each iteration and subtracted from the mean. Then, the model is optimized using stochastic gradient descent (SGD) algorithm to control overfitting, and the model was iterated through 100 cycles with an initial learning rate of 0.225 and momentum of 0.9. A fixed-step decay was used, and the learning rates decayed to 0.0225 and 0.00225 when the number of iterations was 30 and 60, respectively.

### Feature visualization

In the CNN model, the class activation map (CAM) shows discriminative image regions that help in classification [31–33]. The last convolutional layer of the neural network contains the richest spatial and semantic information; therefore, the CAM makes full use of the last convolutional layer features and replaces the later fully connected and softmax layers with a global average pooling (GAP) layer, replacing the values of the entire feature map with the mean values of all pixels. Each feature map has a corresponding weight, and the weighted sum of the globally averaged pooled feature map provides the class activation thermodynamic diagram of the corresponding category and corresponding prediction scores.

### Influence of different multiples

Previous studies trained microscopic images of the three sections and then identified the test images [1, 34]. Here, we explored the impact of different multiples on identification results and feature visualization. This experiment selected the cross-sections of three species, *Carapa guianensis*, *D. latifolia,* and *P. indicus* with 2.5 × and 5 × images in the dataset to discuss the relationship between shooting magnification and visualization results. These three tree species were selected because images of different magnifications were collected under the same experimental conditions, enabling comparative analyses to be conducted. Both datasets were cropped to 800 × 800 simultaneously. The overlap rate of each block patch was approximately 20%. Owing to the small number of training categories, the final accuracy was 100% after 100 iterations for the ResNet152 model.

## Results and discussion

### Identification results of ResNet152 and the accuracy against the patch size

**Fig. 3:**
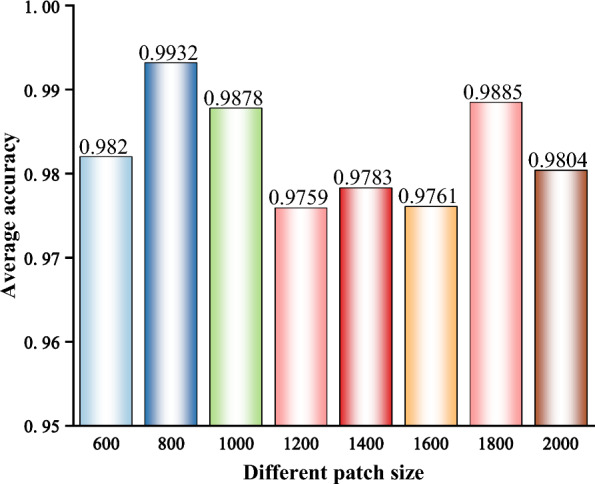
The classification accuracy against different patch sizes

Before training, different patch sizes were first extracted at a repetition rate of 20%, and the tree species were recognized using ResNet152. The accuracy is shown in Fig. 3. The highest classification accuracy (0.9932) was achieved with a patch size of 800 × 800 pixels. Although the ResNet152 classification accuracy was highest among the results, the 800 × 800 pixel patch size was too small to cover all the wood anatomical features. Therefore, the model was trained with a larger 1800 × 1800 pixel patch size for the feature visualization experiments. The results of four closely related species, *P. indicus*, *P. macrocarpus*, *P. soyauxii,* and *P. tinctorius* were selected for discussion.

This result demonstrates that ResNet152 can accurately identify the selected tree species in the dataset. In existing study, CNN was used to identify cross-section microscopic images of 112 tree species, and the overall accuracy reached 95.6% [34]. Our study showed a relatively higher accuracy despite the difference of the image datasets and models deployed.

**Fig. 4:**
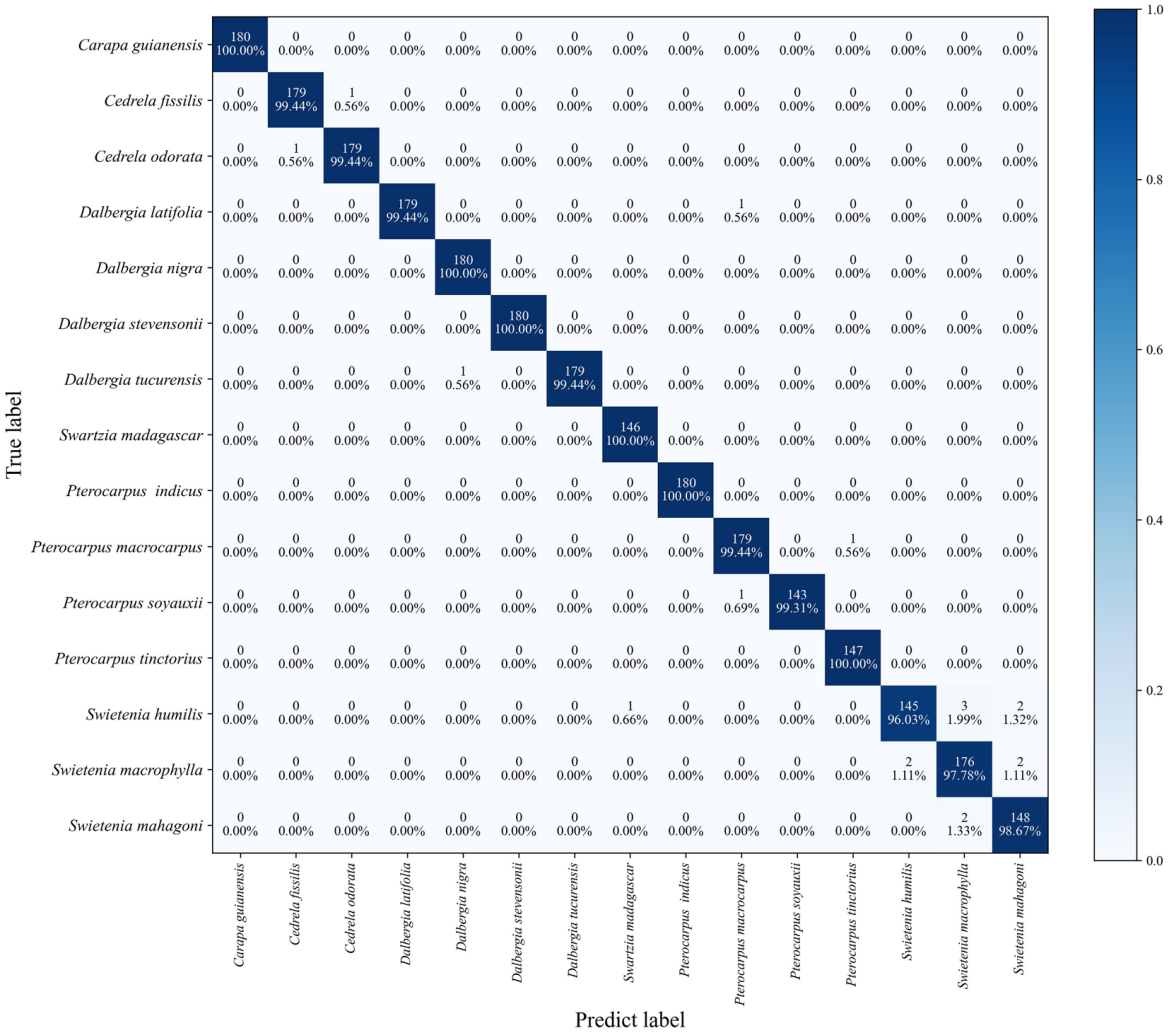
Classification result confusion matrix

The confusion matrix of the classification results when using the 800 × 800 patches are shown in Fig. 4. The number of misidentified patches of these species was less than one except for *Swietenia mahagoni*, *Swietenia humilis,* and *Swietenia macrophylla.* Three patches of *Swietenia humilis* were misidentified as *Swietenia macrophylla*. Two patches of *Swietenia macrophylla* were misidentified as *Swietenia mahagoni* and two patches were misidentified as *Swietenia humilis*. Only two patches of *Swietenia mahagoni* were misidentified as *Swietenia macrophylla*. Overall, the number of erroneous patch sizes in the test set was 17 with a classification accuracy of 99.32%.

Only two *Dalbergia* images were incorrectly identified in the confusion matrix of the classification results. Simultaneously, within the *Pterocarpus* genus, species exhibit similar characteristics. *P. tinctorius* and *P. soyauxii* are listed in CITES Appendix, while the other two species are not listed in CITES Appendix. Therefore, obtaining more accurate identification results is necessary to combat illegal logging. Only two cases of images of *Pterocarpus* were identified incorrectly in the classification results. And one case of CITIES-listed *P. soyauxii* was incorrectly identified as non-CITES *P. macrocarpus*.

The features of the macroscopic images were significantly similar, thus, it is difficult for deep-learning models to determine the differences between the features of different species. By contrast, the features of the microscopic images were finer, and the differences between tree species can be represented by pixels. As a result, deep-learning models can easily reach correct identifications. Although differences can be identified through traditional wood anatomy, deep-learning methods are automatic and timesaving.

Identifying similar features using quantitative wood anatomy data coupled with machine learning analysis has become a common method, making it easier to distinguish key features among species. However, current studies have only focused on improving accuracy and has not been able to explain the specific features used for classification [34]. This study demonstrates that the combination of deep learning with microscopic images yields better performance [14, 35] and further provides the explanation deep-learning classification results by feature visualization.

### Feature visualization

**Fig. 5:**
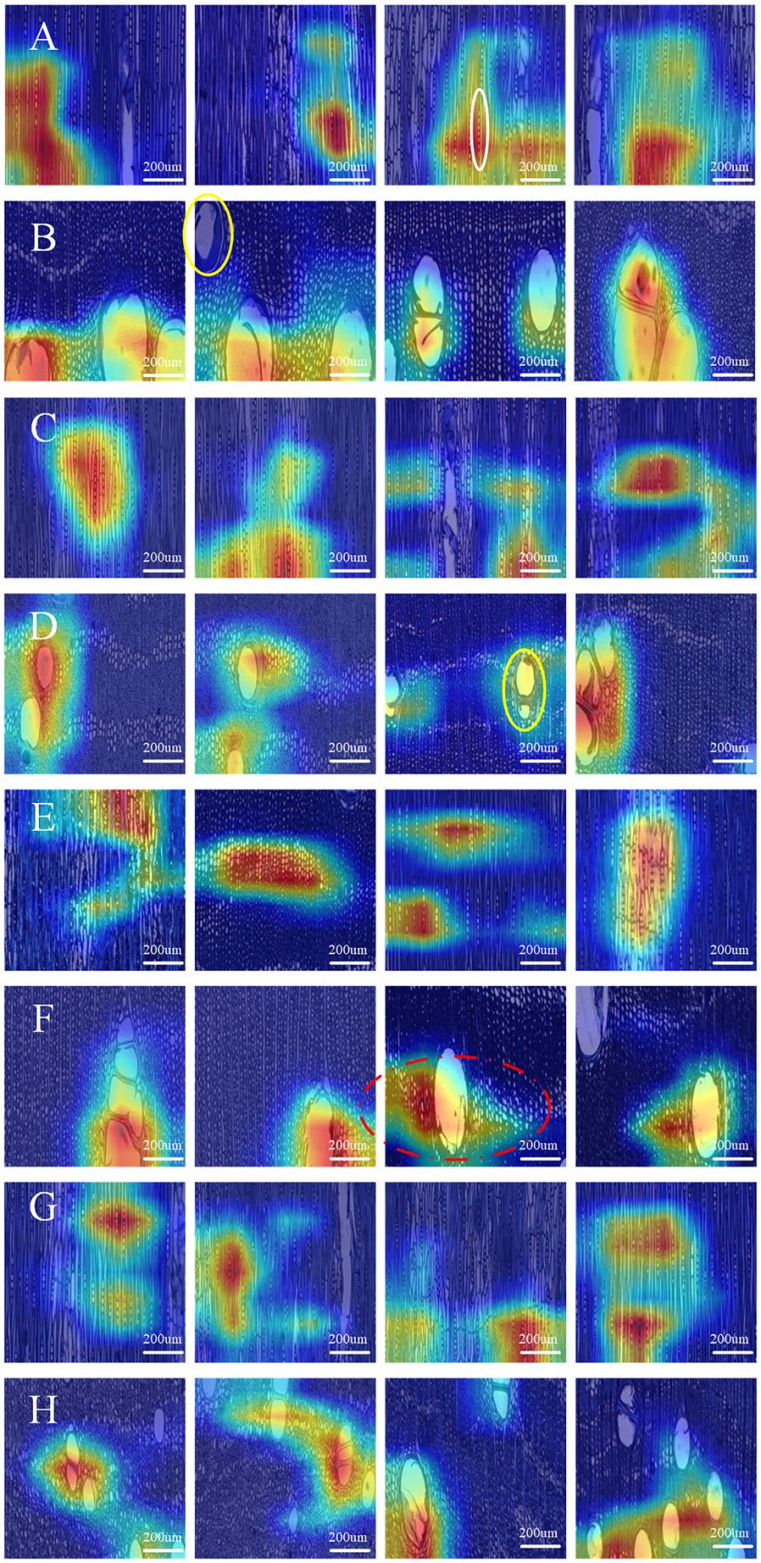
Feature visualization results of *Pterocarpus.* **A**, **B** *P. indicus* in tangential section and cross section; **C**, **D** *P. macrocarpus* in tangential section and cross section; **E**, **F** *P. soyauxii* in tangential section and cross section; **G**, **H** *P. tinctorius* in tangential section and cross section. The yellow circles represent vessels that were not activated by the model. The red dashed circles represent axial parenchyma near the vessels were activated by the model. The white circle represents wood ray in tangential section were actvated by the model

Although the classification accuracy of the patch size of 800 × 800 pixels was the highest among the classification results of ResNet152, considering that the patch size of 800 × 800 pixels was too small to cover all wood anatomical features, the model trained with a patch size of 1800 × 1800 pixels was selected for the feature visualization experiments. The results of four closely related species, namely *P. indicus*, *P. macrocarpus*, *P. soyauxii,* and *P. tinctorius* were selected for discussion. The results are shown in Fig. 5.

*P. indicus* and *P. macrocarpus* have similar anatomical features; specifically, the vessel diameter of *P. indicus* is always larger than *P. macrocarpus* [36]. According to Insidewood [37], the axial parenchyma arrangements of all *Pterocarpus* species are aliform banded and terminal. The ray widths and heights of *P. indicus* and *P. macrocarpus* showed few differences, compared with those of *P. soyauxii* and *P. tinctorius*. The wood rays of *P. indicus* and *P. macrocarpus* can be considered similar to those of *P. soyauxii* and *P. tinctorius.* Both groups can be classified based on the width and height of the wood rays [15].

As shown in Fig. 5, the key features of the four species of *P. indicus*, *P. macrocarpus*, *P. soyauxii,* and *P. tinctorius* are shown in the cross section as axial parenchyma arrangements near the vessel and in the tangential section as the distribution of wood rays. This indicates that the results of feature visualization were consistent with the results of traditional wood anatomy. Some key features are lost during model training as the input images undergo a down-sampling process. The classifier did not visualize all features, including the vessels, axial parenchyma, and wood rays, which may affect the feature results.

### Visualization results for different multiples

**Fig. 6:**
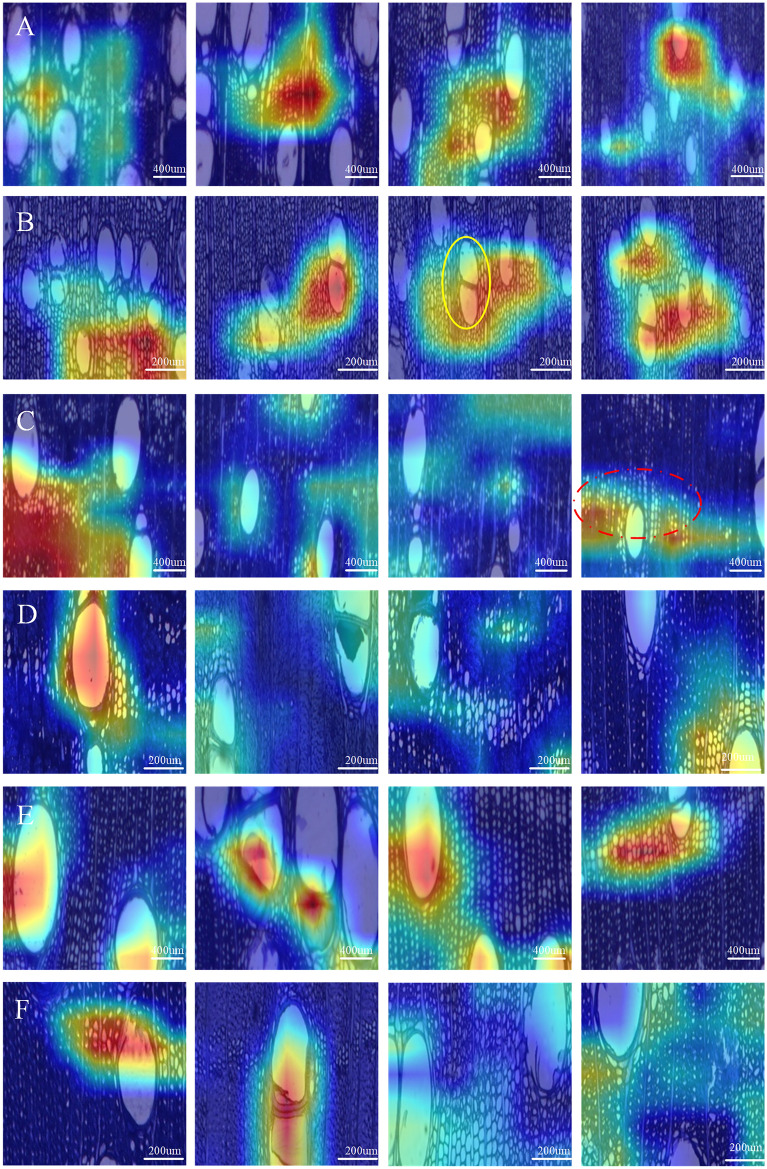
Visualization Results of *Carapa guianensis*, *D. latifolia* and *P. indicus* at different multiples. **A** *Carapa guianensis* at 2.5 × ; **B** *Carapa guianensis* at 5 × ; **C** *D. latifolia* at 2.5 × ; **D** *D. latifolia* at 5 × ; **E** *P. indicus* at 2.5 × ; **F** *P. indicus* at 5 × . The yellow circles represent vessels that were not activated by the model. The red dashed circles represent axial parenchyma near the vessels were activated by the model

Although our previous study conduct feature visualization of deep learning models with macroscopic images of *Dalbergia* and *Ptercarpus* species, images from different multiples were not tested, which is especially important for microscopic images [19]. The visualization results for *Carapa guianensis* (Fig. 6), showed different multiples had no effect on activated features, specifically for the axial parenchyma arrangements near the vessels. The computer considered the arrangement of the axial parenchyma as the main factor for identification, with vessels also having some influence on the identification results. Based on this result, we consider that using 5 × images are much better because they can balance the number of images with the field of view and ensure the integrity of the organizational features within the image.

### Identification results of the vessel dataset

**Fig. 7:**
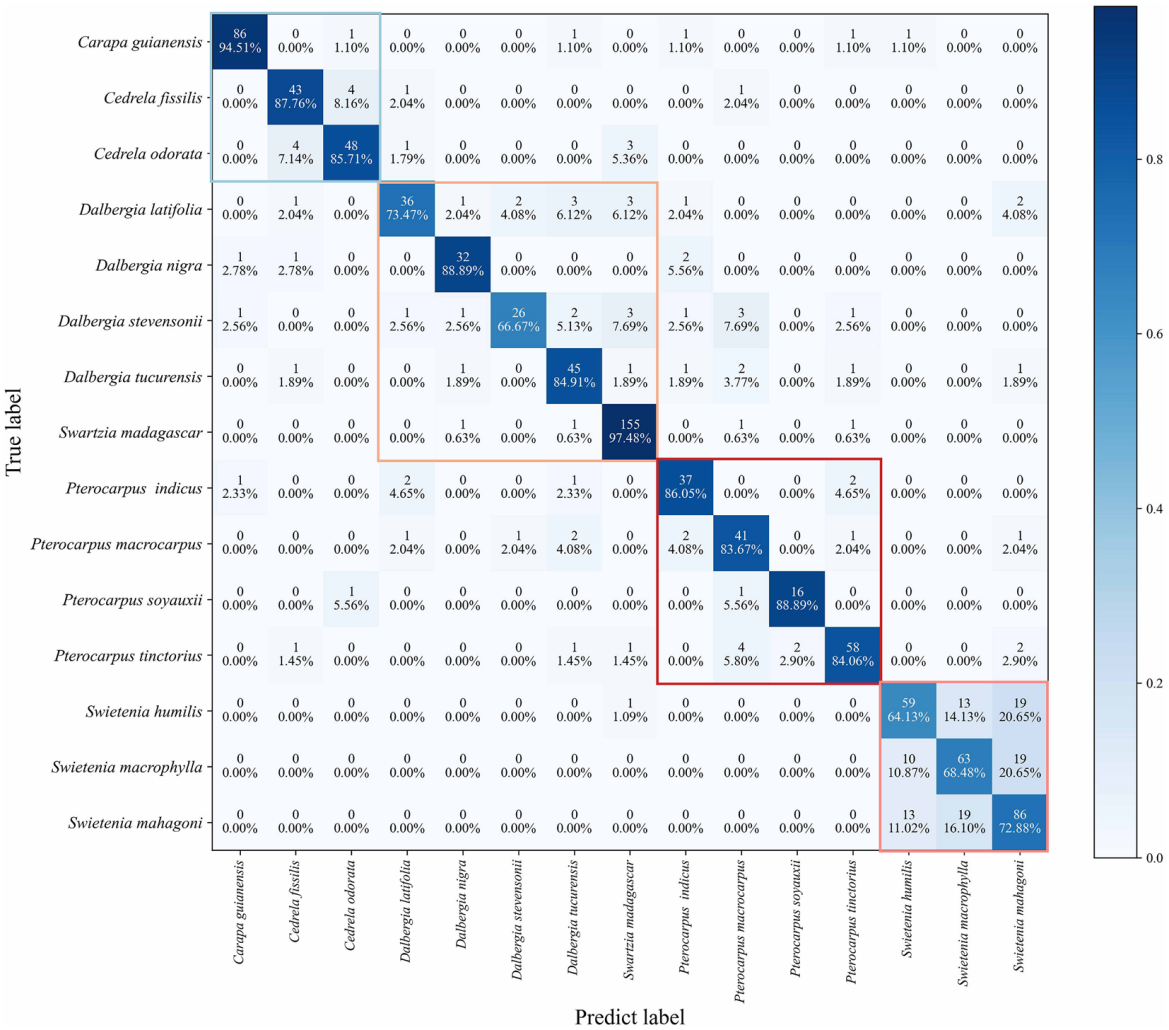
Confusion matrix diagram of vessel dataset. The different color boxes represent tree species that belong to Group 1–4

As shown in Fig. 7, the vessel dataset was modeled, the performance of the network was evaluated, and the final confusion matrix was obtained with an average precision of 83.15% for each tree species. Only *Carapa guianensis* and *Swartzia madagascar* had higher classification accuracies of 94.51% and 97.48%, respectively, indicating that the model could identify these species only by vessels. This suggests that vessel features have a greater influence on species identification.

In contrast, the classification accuracies of *Swietenia humilis*, *Swietenia macrophylla*, and *Swietenia mahagoni* were 64.13%, 68.48%, and 72.88%, respectively, indicating the relatively small influence of vessels. Interestingly, the vessels of all three *Swietenia* species were similar. Although it had low classification accuracy, none of the *Swietenia* species were identified as *Pterocarpus*, *Dalbergia*, *Cedrela*, *Carapa,* or *Swartzia,* and the differences among these three species were small. It also confirms the conclusion that “the wood of the *Swietenia* species cannot be separated anatomically with any degree of certainty” [38]. For similar species of *Carapa guianensis* and *Cedrela odorata*, only two cases of test examples existed in the dataset, where *Cedrela odorata* was incorrectly identified as *Carapa guianensis*, and for these species, the model could differentiate based on the vessels.

Among the four species of *Dalbergia*, the classification accuracies for *D. latifolia* and *D. stevensonii* were lower (73.47% and 66.67%, respectively). However, within the same genus, there were fewer misidentifications, with only *D. latifolia* and *D. stevensonii* being more easily misidentified. This indicates that the vessels are one of the main identification features of *Dalbergia*. This result is consistent with “main wood anatomical features activated by the model for *Dalbergia* species were mainly vessel groupings” [18].

Among the four species of *Pterocarpus*, the classification accuracy of the vessel data was relatively high compared to that of *Dalbergia*, all of which were greater than 80%. However, there were few misidentifications among these four species. This indicates that vessels are one of the main features of *Pterocarpus,* which is consistent with “the deep-learning model was more sensitive to the axial parenchyma arrangement than to the vessel groupings and other anatomical features” [19].

Except for the three *Swietenia* species, *Dalbergia latifolia*, and *Dalbergia stevensonii*, the accuracy of vessel classification for the remaining 10 species ranged from 83.76 to 97.48%, and only a few examples of misidentified species, indicating that for these 10 species, vessel features had a greater influence on wood identification but could not be used as a final basis for discrimination.

## Conclusion

Traditional in situ screening of wood species relies on wood anatomists using hand lenses, which is a time-consuming method that usually identifies only to the genus level, whereas existing intelligent classification methods fail to provide a basis for judgments. In this study, we developed a deep-learning model to identify microscopic images of similar tree species and screen the key features among these species. Images of 15 species were collected from the cross and tangential sections of wood specimens, and the ResNet152 model trained on the images achieved a classification accuracy of 99.3%, indicating a more accurate overall performance than that of wood anatomists. The key features between species were targeted by class activation maps, and the results showed that the key features were axial parenchyma arrangements near the vessel in the cross-sectional images, and the distribution of wood rays was shown in the tangential section. In species identification, it has been proven that different magnifications do not affect species identification or visualization. Moreover, the degree of importance of vessel features in cross-sectional images for different species in depth model identification was determined, and the results showed that vessels were among the main features of *Dalbergia, Pterocarpus*, *Swartzia*, *Carapa,* and *Cedrela*. The research results provide a computer-assisted tool for identifying endangered tree species and present visible identification results for judgment, which can be used to combat illegal logging and related trade and contribute to the implementation of CITES regulations and the conservation of global biodiversity.

### Supplementary Information

**Additional file 1. ** Microscopic anatomically features for selected species showing their similarity of each group.

**Additional file 2.** The detailed information of selected species in this study.

## Data Availability

The scripts used for classification in this study are available on Github: https://github.com/zhengchang01/woodslice-classification-based-ResNet152/tree/master.
