# Supplementary material for: Opening the black box: explainable deep-learning classification of wood microscopic image of endangered tree species
Source: Plant Methods. 2024 Apr 24;20:56. doi: 10.1186/s13007-024-01191-6 (PMC11044446; doi:10.1186/s13007-024-01191-6)
Supplement: Supplementary file 2 — Additional file 2. The detailed information of selected species in this study. [file 13007_2024_1191_MOESM2_ESM.pdf]

Opening the black box: Explainable deep-learning classification of wood microscopic image of endangered tree species

Chang Zheng[1,2], Shoujia Liu [1,2], Jiajun Wang [1,2,4], Yang Lu [1,2], Lingyu Ma [1,2], Lichao Jiao [1,2], Juan Guo [1,2], Yafang Yin [1,2], Tuo He [1,2,3]*

1 Department of Wood Anatomy and Utilization, Research Institute of Wood Industry, Chinese Academy of Forestry, Beijing 100091, China

2 Wood Collections, Chinese Academy of Forestry, Beijing 100091, China

3 Wildlife Conservation Monitoring Center, National Forestry and Grassland Administration, Beijing 100714, China

4 National Centre for Archaeology, Beijing 100013, China

Table 1 Protection level, number of wood specimens and collected images for selected species

Class label	Species	Protection level	Number of specimens	Number of labeled vessels
**1**	*Carapa guianensis*	-	7	193
**2**	*Cedrela fissilis*	CITES II	6	234
**3**	*Cedrela odorata*	CITES II	10	262
**4**	*Dalbergia latifolia*	CITES II	6	215
**5**	*Dalbergia nigra*	CITES I	6	173
**6**	*Dalbergia stevensonii*	CITES II	7	185
**7**	*Dalbergia tucurensis*	CITES II	6	243
**8**	*Swartzia madagascar*	-	5	305
**9**	*Pterocarpus indicus*	-	6	198
**10**	*Pterocarpus macrocarpus*	-	6	217
**11**	*Pterocarpus soyauxii*	CITES II	5	88
**12**	*Pterocarpus tinctorius*	CITES II	5	277
**13**	*Swietenia humilis*	CITES II	9	241
**14**	*Swietenia macrophylla*	CITES II	15	257
**15**	*Swietenia mahagoni*	CITES II	12	321

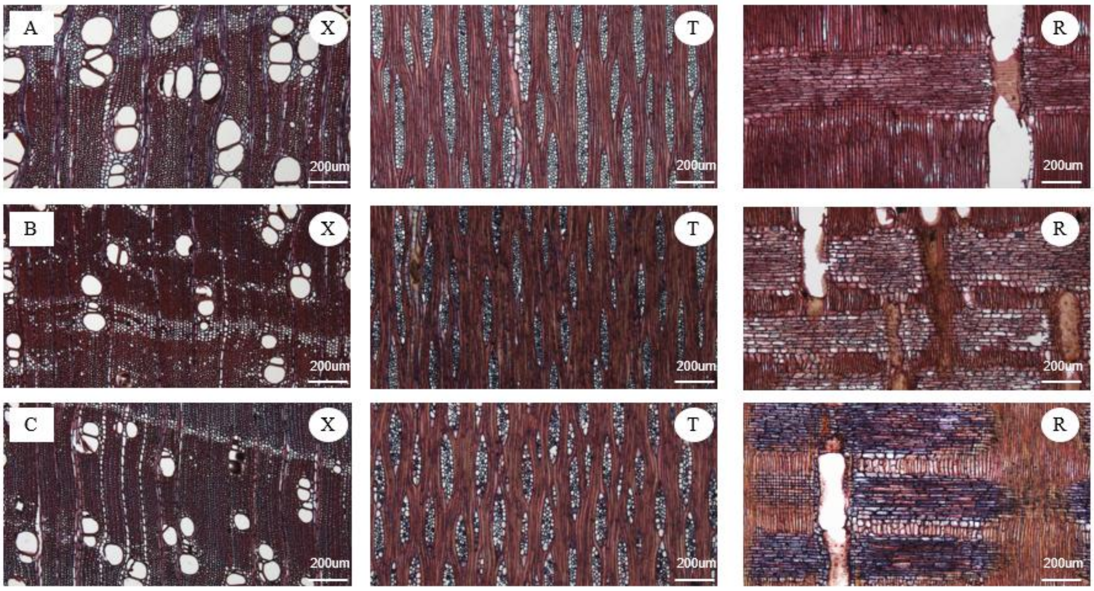

Figure S1 Light micrographs of the woods included in Group 1. X-transverse, T-tangential, and R-radial sections of *Carapa guianensis* (A), *Cedrela_fissilis* (B), *Cedrela odorata* (C) showing the similarity of the three species concerning wood anatomical features.

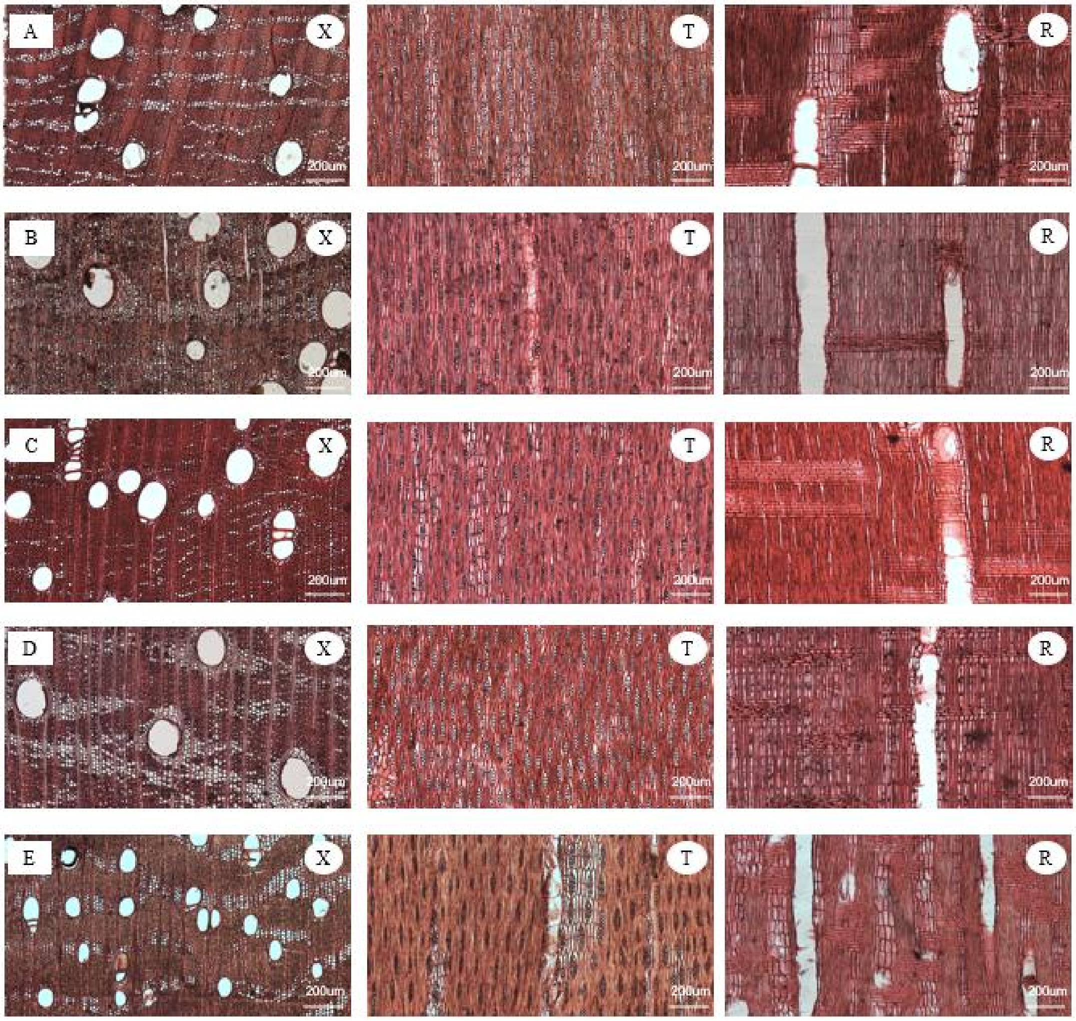

Figure S2: Light micrographs of the woods included in Group 2. X-transverse, T-tangential, and R-radial sections of *Dalbergia latifolia* (A), *Dalbergia nigra* (B), *Dalbergia stevensonii* (C), *Dalbergia tucurensis* (D), *Swartzia madagascar* (E) showing the similarity of the five species concerning wood anatomical features.

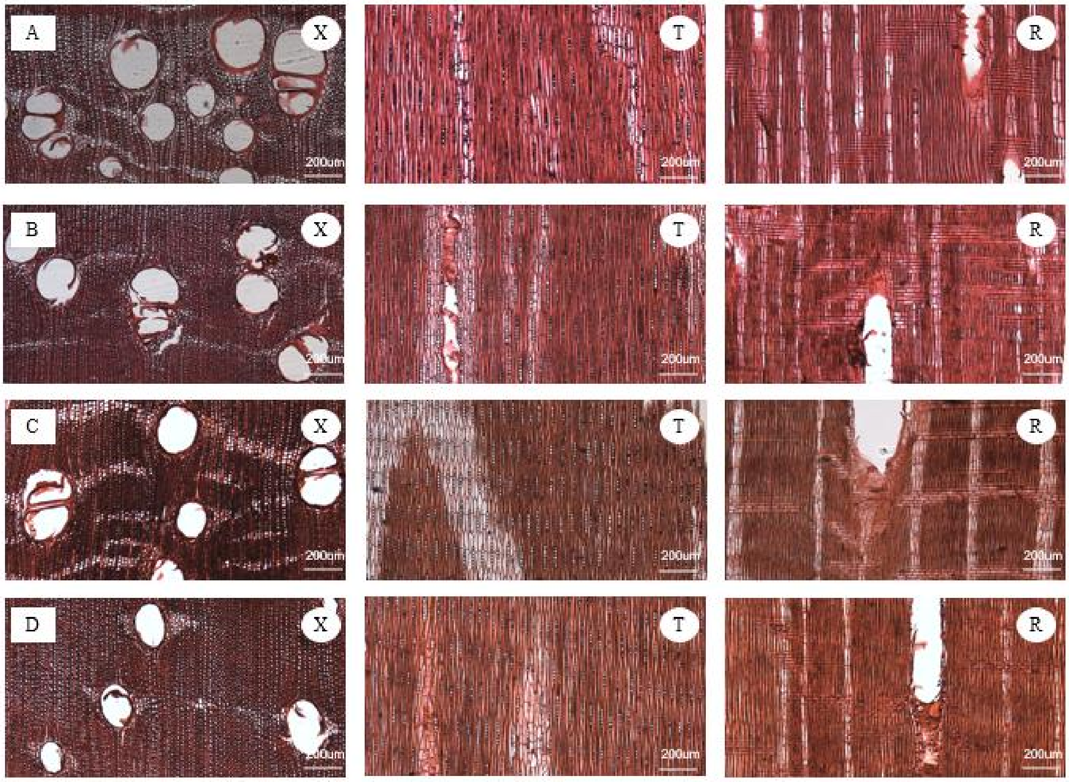

Figure S3: Light micrographs of the woods included in Group 3. X-transverse, T-tangential, and R-radial sections of *Pterocarpus indicus* (A), *Pterocarpus macrocarpus* (B), *Pterocarpus soyauxii* (C), *Pterocarpus tinctorius* (D) showing the similarity of the four species concerning wood anatomical features.

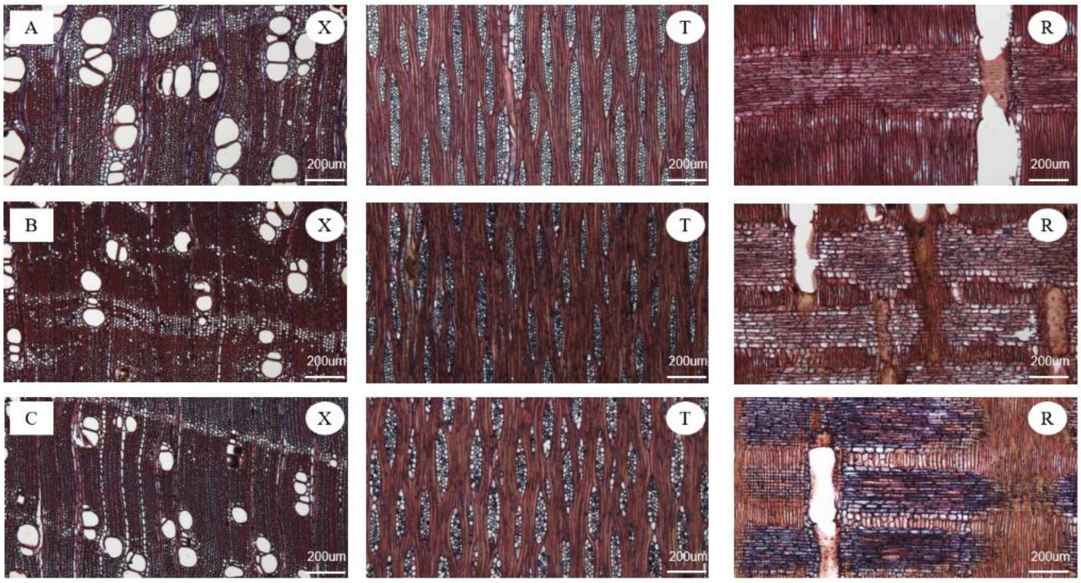

Figure S4: Light micrographs of the woods included in Group 4. X-transverse, T-tangential, and R-radial sections of *Swietenia macrophylla* (A), *Swietenia_humilis* (B), *Swietenia_mahagoni* (C) showing the similarity of the three species concerning wood anatomical features.
